# Familial and socioeconomic contributions to premorbid functioning in psychosis: Impact on age at onset and treatment response

**DOI:** 10.1192/j.eurpsy.2020.41

**Published:** 2020-04-29

**Authors:** Alex Hatzimanolis, Pentagiotissa Stefanatou, Emmanouil Kattoulas, Irene Ralli, Stefanos Dimitrakopoulos, Stefania Foteli, Ioannis Kosteletos, Leonidas Mantonakis, Mirjana Selakovic, Rigas-Filippos Soldatos, Ilias Vlachos, Lida-Alkisti Xenaki, Nikolaos Smyrnis, Nicholas C. Stefanis

**Affiliations:** 1 Department of Psychiatry, National and Kapodistrian University of Athens Medical School, Eginition Hospital, 11528 Athens, Greece; 2 Neurobiology Research Institute, Theodor-Theohari Cozzika Foundation, 11521 Athens, Greece; 3 University Mental Health, Neurosciences and Precision Medicine Research Institute, 11527 Athens, Greece

**Keywords:** Family history, first-episode psychosis, premorbid adjustment, schizophrenia, socioeconomic status, treatment response

## Abstract

**Background.:**

Premorbid adjustment (PA) abnormalities in psychotic disorders are associated with an earlier age at onset (AAO) and unfavorable clinical outcomes, including treatment resistance. Prior family studies suggest that familial liability, likely reflecting increased genetic risk, and socioeconomic status (SES) contribute to premorbid maladjustment. However, their joint effect possibly indicating gene–environment interaction has not been evaluated.

**Methods.:**

We examined whether family history of psychosis (FHP) and parental SES may predict PA and AAO in unrelated cases with first-episode psychosis (*n* = 108) and schizophrenia (*n* = 104). Premorbid academic and social functioning domains during childhood and early adolescence were retrospectively assessed. Regression analyses were performed to investigate main effects of FHP and parental SES, as well as their interaction. The relationships between PA, AAO, and response to antipsychotic medication were also explored.

**Results.:**

Positive FHP associated with academic PA difficulties and importantly interacted with parental SES to moderate social PA during childhood (interaction *p* = 0.024). Positive FHP and parental SES did not predict differences in AAO. Nevertheless, an earlier AAO was observed among cases with worse social PA in childhood (*β* = −0.20; *p* = 0.005) and early adolescence (*β* = −0.19; *p* = 0.007). Further, confirming evidence emerged for an association between deficient childhood social PA and poor treatment response (*p* = 0.04).

**Conclusions.:**

Familial risk for psychosis may interact with parental socioeconomic position influencing social PA in childhood. In addition, this study supports the link between social PA deviations, early psychosis onset, and treatment resistance, which highlights premorbid social functioning as a promising clinical indicator.

## Introduction

For over two decades, the neurodevelopmental hypothesis for schizophrenia (SZ) etiology is the most prominent conceptualization for SZ pathogenesis, supporting the occurrence of early life brain-related developmental abnormalities which might be caused by both genetic and environmental determinants [1–3]. Premorbid adjustment (PA) difficulties likely represent an index of neurodevelopmental compromise and have been recognized as a significant risk factor for the development of SZ later in life [4–6]. Longitudinal studies in clinical high-risk populations have provided evidence for increased conversion rates to psychosis among individuals with PA deficits [7–9], while poor social PA has been associated with prodromal signs of psychosis [10]. Impairments in both social and academic PA have been extensively documented in adult patients with SZ [6, 11–17], bipolar disorder [11,18], and patients with first-episode psychosis (FEP) [19,20]. Importantly, PA abnormalities among patients with psychotic disorders, particularly social maladjustment, may represent a reliable predictor of unfavorable long-term clinical outcomes and enduring negative symptoms [21–27], cognitive dysfunction [28], and inadequate treatment response [29–31].

Previous research has shown that individuals with early-onset SZ are characterized by more pronounced PA deficits than adult-onset cases [32], implying that neurodevelopmental disruptions are responsible for the earlier expression of psychotic symptoms, possibly attributed to genetic risk factors [33]. A suspected genetic component may be at least in part responsible for the PA deficits observed in patients diagnosed with SZ [6,34]. Few studies have demonstrated an association between poor PA and the existence of positive family history for psychiatric illness among individuals with SZ [35,36], yet opposing findings have also been reported [37]. In addition, a number of family studies have documented PA weaknesses in relatives or unaffected siblings of individuals with SZ [6,18,38,39], indicating that genetic risk for SZ may be a contributing factor to premorbid dysfunction. Prior evidence indicate that poor PA could be related with an earlier age at onset (AAO) in individuals experiencing psychosis [20,21,32,37], suggesting that early life neurodevelopmental aberrations might worsen the progression of the clinical syndrome and accelerate the onset of psychotic symptoms. In support of the above view, observations in patients with early-onset SZ and bipolar disorder suggest the involvement of neurodevelopmental pathways in the development of psychosis [40,41]. Familial effects have been demonstrated for AAO in psychotic disorders [37, 42,43]; however, negative findings also exist [44,45].

Premorbid maladjustment is postulated to reflect disrupted neurodevelopmental trajectories [20], likely predisposing a subgroup of vulnerable individuals to develop psychotic symptoms lying in the SZ-spectrum or affective psychoses diagnostic entities [11,12] and is often documented among first-episode psychotic patients [21–23]. Herein, we aimed to amalgamate and analyze demographic and clinical data collected from well-characterized unrelated cases diagnosed with FEP and SZ in an attempt to investigate the potential moderating role of familial liability to psychosis and parental socioeconomic status (SES) on aspects of PA and AAO. Family history of psychosis (FHP) is considered a proxy phenotype of genetic risk [46,47], while parental SES denotes an index of socioeconomic position associated with PA [48] and represents an environmental risk factor for psychosis-spectrum disorders [49–51] that might interact with genetic liability to further increase the likelihood of developing SZ [47,52]. Prompted by recent evidence implicating deviant PA and early AAO in treatment resistance in SZ [31], early response to antipsychotic medication in FEP cases and treatment resistance in SZ cases were also evaluated.

## Methods

### Participants

#### First-episode psychosis patients

A total of 130 FEP cases (mean age: 25.5 ± 7.3 years; age range: 16–45 years) were assessed in five mental health services throughout the metropolitan area of Athens, as part of an ongoing longitudinal research project aiming to investigate the involvement of genetic and environmental determinants on psychosis risk. National legislation dictates that adult mental health care and hospitalization is provided to all patients above the age of 16 years. Sample eligibility criteria and detailed clinical information are reported elsewhere [53]. Exclusion criteria included: (a) age at psychosis onset <16 years, (b) the presence of psychotic symptoms due to an organic cause or acute intoxication, (c) history of serious neurological disorder, (d) intelligence quotient <70, and (e) belonging to the ultra-high-risk phenotype [54]. At admission, 75% of cases were either drug-naïve or had received medication for a single day (Table S1). All cases were screened using the Diagnostic Interview for Psychoses (DIP) [55], a standardized semi-structured interview which generates diagnoses according to different diagnostic algorithms, on the basis of the Operational Criteria Checklist for Psychotic Illness (OPCRIT) [56]. According to the International Classification of Diseases 10th Revision (ICD-10), 83.2% of cases were diagnosed with nonaffective psychotic disorder (ICD-10 codes: F20-29). Written informed consent was obtained after a detailed description of the research objectives and the study protocol was approved by the Institutional Review Board at Eginition University Hospital.

#### Schizophrenia patients

We recruited 115 male inpatients (mean age: 33.2 ± 8.6 years; age range: 19–50 years) who met the Diagnostic and Statistical Manual of Mental Disorders, 4th edition (DSM-IV) diagnostic criteria for SZ (American Psychiatric Association, 1994). SZ cases were examined by trained psychiatrists (E.K., N.S.) and a clinical psychologist (P.S.) during their hospitalization at the secured psychiatric ward for adult male individuals in Eginition University Hospital (Athens, Greece). All patients were clinically evaluated using the DIP diagnostic interview [54] and at the time of assessment were receiving drug treatment with either conventional or atypical antipsychotics (Table S1). Among cases, the mean years of illness were 11.2 ± 8.5. Exclusion criteria included (a) the presence of mental retardation, (b) history of serious neurological disorder, (c) illness onset preceding completion of the 16th year of age, and (d) unavailability of close relatives to provide valid information related to premorbid functioning. All patients provided signed informed consent before entering the study.

### Assessments

#### Premorbid adjustment

The Cannon-Spoor Premorbid Adjustment Scale (PAS) [57] was administered to assess PA in both SZ and FEP cases. The PAS retrospectively examines aspects of premorbid functioning across four developmental stages: childhood (up to 11 years), early adolescence (12–15 years), late adolescence (16–18 years), and adulthood (19 years and beyond); and across two domains: academic (scholastic performance, adaptation to school) and social (sociability/withdrawal, peer relationships, and socio-sexual functioning). Detailed information with regard to early life functioning was obtained by trained psychiatrists via semi-structured interviews with both patients and their family members (i.e., parents or siblings). Social PA was estimated through the items of peer relationships and sociability/withdrawal at each age period. Academic PA was estimated through the items of scholastic performance and school adaptation at each developmental stage. All cases included in this study had an illness onset >16 years and completed only childhood and early adolescence PAS subscales to minimize the possibility that behavioral aspects related to the prodromal phase of the illness are captured [15]. In accordance with previous studies, premorbid period was defined as ending 1 year before the emergence of positive psychotic symptoms [58,59]. For each developmental stage, academic and social domain PAS scores were estimated, with higher score denoting poorer PA.

#### Socioeconomic status

Parental SES classification was based on available information related to parental academic achievement and occupational status, as well as total household income per year. Both cases and their accompanying relatives (mainly parents) were interviewed in order to obtain reliable information. Three SES groups (i.e., high, middle, and low) were generated as follows: High SES group included cases with high parental educational level (university or college degree) and occupational activity (i.e., professionals, senior officials, high grade employees, managers), while the annual family gross income exceeded the average family gross income reported for the years 2011–2017 by the Hellenic Statistical Authority (ELSTAT); middle SES group included cases with intermediate level of parental education (high-school terminated, technical school diploma) and occupation status (i.e., low-level employee, technician), while the annual family gross income was similar or slightly lower from the country’s average (up to 20% below national average) reported by ELSTAT; low SES group included the remaining cases and those cases that reported family gross income equal or below the country’s poverty threshold, irrespective of the parental educational/occupational level. Valid SES data were obtained from 202 cases (100 FEP; 102 SZ), as a small number of cases or family members denied to provide relevant information or their responses deemed untrustworthy.

#### Family history

The occurrence of psychotic illness among biological relatives was determined using the Family Interview for Genetic Studies [60]. Cases reporting that a first or second degree relative was diagnosed with affective or nonaffective psychotic disorder were marked as having positive FHP (details in supplementary text). Positive FHP has been considered a proxy phenotype for increased genetic risk of psychosis [46,47], supported by molecular genetic evidence demonstrating that individuals with positive FHP are characterized by higher polygenic loading for SZ [61,62].

#### Age at onset

In both patient groups, we recorded the age at which psychotic symptoms (principally positive symptoms) appeared for the first time (OPCRIT item 4), as part of the DIP diagnostic interview [55] on the basis of information gathered from both the patient and his/her family members. Cases with an AAO greater than 16 years were considered for further analysis.

#### Response to treatment

Administration of clozapine (34 SZ cases) was used as a proxy measure of treatment resistance in SZ cases [31]. Treatment response assessment in FEP cases was based on Positive and Negative Syndrome Scale (PANSS) scores obtained at admission (baseline assessment) and 4 weeks later (follow-up assessment). Symptomatic remission was determined according to the Andreasen’s consensus remission criteria [63]. Cases not fulfilling the above criterion for clinical remission were defined as nonresponders to antipsychotic medication (36 FEP cases). Prior evidence suggests that greater than 80% of FEP cases who do not initially respond to first-line antipsychotic treatment will develop treatment resistance [64].

### Statistical analyses

Socio-demographic and clinical characteristics were compared between FEP and SZ patient groups using *χ* ^2^-tests for dichotomous variables and analyses of variance for continuous variables. Linear regression analyses were performed to assess the impact of FHP status (negative FHP set as reference) and parental SES (high parental SES set as reference) on PA domain scores and AAO, adjusting for gender, age, and psychiatric site at admission. Further, as the analyses were conducted in a mixed sample of FEP and SZ cases, we adjusted all models for diagnostic status, while separate analyses were performed in each patient group. PA domain scores were standardized before analysis and a log-transformation was applied to AAO to account for positive skewness. The association between PA domain scores and AAO was examined using linear regressions and logistic regressions were performed to test associations with treatment resistance, adjusting for the same covariates as above. Sensitivity analyses were also performed to account for possible confounding effect related to medication exposure at baseline. Multiple regression models were fitted to investigate interaction effects between FHP and parental SES status on PA domains, AAO and treatment resistance, including both the main effects of each individual predictor (FHP, parental SES) and their interaction term. An interaction on the additive scale was declared if the estimated effect size of the interaction term was greater than the sum of individual effect sizes of each predictor. It has been suggested that the presence of additive statistical interaction most likely designates a factual biological mechanism [65]. Adjusted *R*-squared (*R* ^2^) estimates were calculated to assess the proportion of the explained phenotypic variance. As in principle, this study aimed to provide external validity to previously reported findings and the analysis of joint effects between predictors (interaction models) was deemed exploratory, the level of statistical significance was set at *p* < 0.05 (two-tailed). All statistical analyses were conducted using R v3.5.0 (http://www.r-project.org).

## Results

### Comparisons between socio-demographic characteristics

In total, 212 cases were included in our analyses (108 FEP, 104 SZ; 174 males, 38 females). Valid information on parental SES was acquired from 202 cases (100 FEP, 102 SZ; 166 males, 36 females). Compared to SZ cases, FEP cases had lower mean age (*F* = 52.5; *p* < 0.0001), more years of education (*F* = 4.92; *p* = 0.028) and a later AAO (*F* = 5.07; *p* = 0.025), while they did not differ in terms of family history for psychosis (FHP) (*χ* ^2^ = 0.582; *p* = 0.446) and parental SES (*F* = 0.965; *p* = 0.327). Our analyses did not provide evidence that cases with positive FHP were characterized by lower parental SES (*χ* ^2^ = 2.24; *p* = 0.135) or an earlier AAO (*F* = 0.00; *p* = 0.985), yet we confirmed an earlier AAO among males (*F* = 14.0; *p* = 0.0002) and a higher prevalence of positive FHP among females (*χ* ^2^ = 6.41; *p* = 0.011). Moreover, the presence of lower parental SES did not significantly correlate with AAO (*F* = 1.20; *p* = 0.275). Females were more likely to exert social PA difficulties in childhood compared to males (*F* = 4.41; *p* = 0.037), whereas no differences were observed in early adolescence (*F* = 1.01; *p* = 0.316). Similarly, FEP and SZ cases did not differ in terms of academic or social PA in both developmental periods (Table S2). Associations between socio-demographic variables in SZ and FEP cases are depicted in Table 1.

**Table 1.** Demographic characteristics of the examined clinical samples.

	FEP cases (*N* = 108)	SZ cases (*N* = 104)	*p*-value
Gender (males/females)	70/38	104/0	n/a
Age in years (mean ± SD)	25.5 ± 7.3	33.2 ± 8.6	<0.0001
Years of education (mean ± SD)	13.4 ± 2.6	12.4 ± 3.9	0.028
Age at onset (mean ± SD)	25.2 ± 7.3	22.6 ± 9.3	0.025
Parental SES (high/middle/low)	41/35/32	35/31/38	0.327
Positive FHP (%)	44.4	30.8	0.446

Abbreviations: FEP, first-episode psychosis; FHP, family history of psychosis; SES, socioeconomic status; SZ, schizophrenia.

### Effects of FHP and parental SES on PA domains and treatment response

In all, 57 cases reported positive FHP and showed worse academic PA during childhood (*β* = 0.16; *p* = 0.034) compared to negative FHP cases (*n* = 155), whereas no noticeable differences in childhood social PA (*β* = 0.03; *p* = 0.683) or early adolescence PA domains were observed, even though a trend was found for academic PA (*β* = 0.14; *p* = 0.062) (Table 2). With regard to parental SES, we classified cases in three separate groups, namely high SES (*n* = 74), middle SES (*n* = 63), and low SES (*n* = 65). There was no evidence for association between parental SES and academic or social PA in childhood and early adolescence. Likewise, neither FHP (OR = 1.15; 95% CI = 0.57–2.31; *p* = 0.706) nor parental SES (OR = 0.86; 95% CI = 0.60–1.24; *p* = 0.424) was associated with response to antipsychotic treatment.

**Table 2.** Mean (±SEM) values for PA domain scores and AAO for all cases after stratification for FHP and parental SES status.

	FHP			SES			
	**Negative**	**Positive**	***β* (*p*-value^a^)**	**High**	**Middle**	**Low**	***β* (*p*-value^a^)**
Childhood PA							
Academic	−0.132 (0.080)	0.212 (0.137)	0.16 (**0.034**)	−0.233 (0.115)	0.023 (0.123)	0.104 (0.122)	0.13 (0.070)
Social	−0.065 (0.076)	−0.002 (0.131)	0.03 (0.683)	−0.083(0.109)	−0.064(0.117)	0.005(0.116)	0.04 (0.586)
Early adolescence PA							
Academic	−0.139 (0.96)	0.130 (0.098)	0.14 (0.062)	−0.233 (0.97)	−0.009 (0.94)	0.054 (0.99)	0.10 (0.167)
Social	−0.133 (0.94)	0.073 (0.090)	0.07 (0.363)	−0.153 (0.91)	−0.118 (0.96)	0.052 (0.92)	0.03 (0.721)
AAO	24.0 (0.54)	22.8 (0.93)	−0.08 (0.245)	22.5 (0.76)	25.4 (0.81)	23.3 (0.81)	−0.06 (0.367)

Abbreviations: AAO, age at onset; FHP, family history of psychosis; PA, premorbid adjustment; SES, socioeconomic status.

a Results from linear regression analyses, adjusted for sex, age, psychiatric site, and diagnosis status. *p*-value <0.05 is indicated in bold.

### Interplay between FHP and parental SES influences social PA in childhood

**Figure 1.**
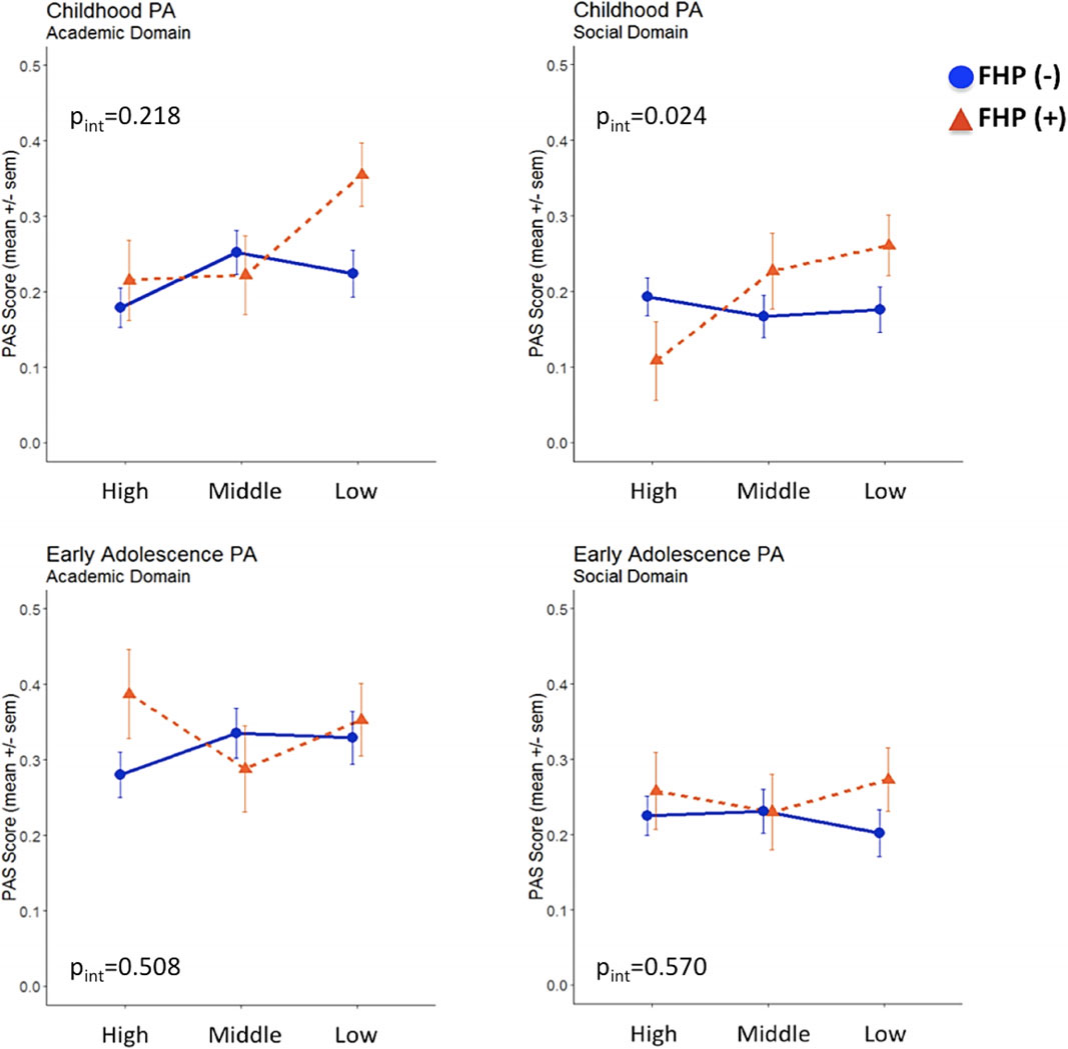
Academic and social premorbid adjustment domain scores in childhood and early adolescence following stratification for family history of psychosis and parental socioeconomic status.

We further fitted multiple linear regression models to test departure from additivity, which would be an indication that FHP and parental SES could jointly impact on PA domains and AAO. A statistically significant additive interaction between FHP and parental SES status on childhood social PA was detected (*β*_interaction_ = 0.194; *p* = 0.024; adjusted *R* ^2^ = 0.021) (Figure 1), as the effect size of the interaction (FHP × SES) term was greater than the sum of the individual effect sizes of FHP and parental SES (Table S1). We observed that cases with both positive FHP and low parental SES reported more pronounced social PA impairments during childhood compared to the remaining cases. Additionally, since a “cross-over” interaction was revealed, cases with positive FHP belonging to the high parental SES subgroup had better social PA within the entire sample, indicating that parental socioeconomic position may moderate early social PA among cases with increased familial loading for psychosis. The interaction effect for academic PA did not reach statistical significance (*β*_interaction_ = 0.106; *p* = 0.218; adjusted *R* ^2^ = 0.003)**.** No evidence for significant FHP × parental SES interactions was detected for early adolescence PA and AAO.

### Deviant social PA predicts an earlier onset of psychosis

We next sought to confirm previous findings indicating that PA abnormalities predispose to an earlier onset of psychotic symptoms [19, 34–36] and further explore the potential moderation by FHP status and parental SES. An inverse correlation was observed between AAO and poor social PA during childhood (*β* = −0.20; *p* = 0.0047) and early adolescence (*β* = −0.19; *p* = 0.0073), whereas academic PA did not significantly impact AAO (Table 3). In subsequent analyses, FHP status and parental SES were added as additional plausible confounders in multiple linear regression models and the association effect sizes were compared with those of the primary unadjusted models. As shown in Table 3, no major differences were detected in the association between AAO and social PA.

**Table 3.** Results of the association between PA domain scores and AAO in FEP and SZ cases.

	FEP cases (*N* = 108)		SZ cases (*N* = 104)		All cases (*N* = 212)		All cases (FHP, SES adjusted)	
	*β*	*p*-value	*β*	*p*-value	*β*	*p*-value	*β*	*p*-value
Childhood PA								
Academic	−0.07	0.496	0.08	0.417	−0.01	0.873	0	0.99
Social	−0.14	0.148	−0.21	0.041^*^	−0.2	0.005^**^	−0.2	0.004^**^
Early adolescence PA								
Academic	−0.23	0.019^*^	0.04	0.723	−0.08	0.264	−0.09	0.241
Social	−0.14	0.156	−0.22	0.040^*^	−0.19	0.007^**^	−0.19	0.008^**^

Abbreviations: AAO, age at onset; FEP, first-episode psychosis; FHP, family history of psychotic disorder; PA, premorbid adjustment; SES, socioeconomic status; SZ, schizophrenia.

* *p* value <0.05.

** *p* value <0.01.

### Evidence implicating poor social PA to treatment resistance

**Figure 2.**
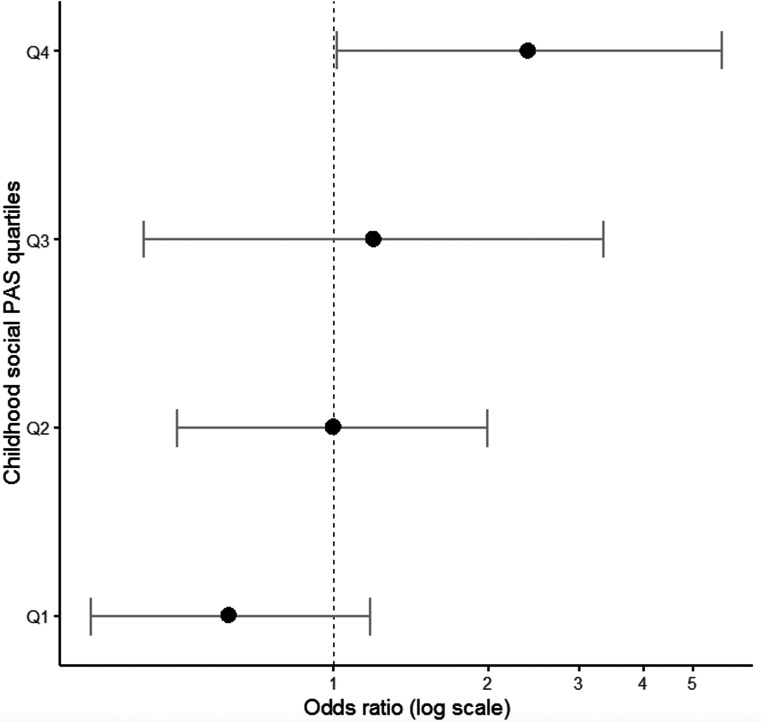
Association between social premorbid adjustment domain score in childhood and treatment response to antipsychotic medication.

To explore the contribution of PA and AAO to antipsychotic treatment response, SZ cases receiving clozapine (treatment resistant cases) and FEP cases not responding to short-term antipsychotic treatment [63] were grouped together (total *n* = 70) forming a nonresponders group of cases and compared with the remaining cases (treatment responders, *n* = 142). It is outlined that treatment response was independent of minimal exposure to medication at baseline (Table S1) in a subset of FEP cases (OR = 1.05; 95% CI: 0.63–1.77; *p* = 0.845). Nonresponders to treatment were characterized by poorer social PA during childhood (OR = 1.37; 95% CI: 0.99–1.89; *p* = 0.059) and an earlier AAO (OR = 0.47; 95% CI: 0.21–1.08; *p* = 0.075), which approached statistical significance. Exploratory analyses showed that cases with the highest (i.e., worse) social PA scores in childhood (upper quartile of the distribution) were more likely to be classified as nonresponders (OR = 2.46; 95% CI: 1.04–5.80; *p* = 0.04) (Figure 2). The above associations remained unchanged following adjustment for FHP, parental SES, and medication exposure at baseline (Table S3). Academic PA in childhood as well as early adolescence PA had no impact on treatment response (all *p* > 0.2).

## Discussion

Our findings provide evidence that familial loading for psychosis, most likely reflecting an increased genetic predisposition [61,62], individually or jointly with an environmental component such as parental socioeconomic background may moderate premorbid functioning among individuals who will later develop a psychotic disorder. Family-based studies have previously reported adjustment difficulties in relatives of psychotic patients, suggesting the involvement of presumed genetic influences [6,18,34,36]. However, no evidence has emerged for synergism between genetic risk and environmental exposures on PA in psychotic disorders, which has been recently implicated in SZ [66]. The present study corroborates the influence of FHP on premorbid academic adjustment [34] and further revealed that parental socioeconomic profile moderates the impact of familial risk for psychosis on early social adjustment. This observation is in agreement with previous studies indicating that familial loading for mental illness and environmental risk factors for psychosis (i.e., urbanicity, social disadvantage, childhood adversity, cannabis use) may act in synergy to increase disease liability [46,51,67–70]. Moreover, population-wide analyses underscore the contribution of upbringing locale, likely related with socioeconomic profile, to psychosis manifestation and the modifiable role of genetic liability in the incidence of psychotic disorders [71].

We observed that individuals with presumably higher genetic risk for psychosis and growing up in families of lower SES could be more vulnerable to social maladjustment in childhood. Epidemiological evidence from Swedish national registers suggests that positive FHP in adopted children may act in synergy with social disadvantage to increase risk for psychotic disorders [52]. Consistent with the above findings, our results imply that genetically predisposed children from families of lower socioeconomic background might exhibit social PA deficits in childhood, which precede the onset of psychotic illness typically in young adulthood. Nevertheless, it is acknowledged that our results do not reveal a causative relationship between poor social PA and the presentation of psychosis, therefore additional validation in independent clinical samples is required to delineate the contribution of G × E on premorbid social functioning. We also note that as a “cross-over” interaction effect was evident, higher parental SES appeared to exert a protective role on social adjustment among cases with positive FHP, indicating that socioeconomic advantage likely increases resilience and eventually prevents the development of PA deviations linked to psychosis expression [72]. It is of interest that recent observations point toward a positive association between downward parental income and greater risk for SZ [73], highlighting parental socioeconomic profile as a key environmental determinant involved in SZ etiology.

Additionally, this study validates a significant correlation between poor social PA and an earlier onset of psychotic symptoms, which has been previously reported in patients diagnosed with FEP or SZ [20,31] and underscores the clinical value of assessing premorbid functioning—particularly social behavior—in the context of early intervention strategies for psychotic disorders in high-risk populations. However, it remains to be determined whether the observed correlation could be attributed to genetic or environmental risk factors. In our cohort, the relationship between social PA and AAO was independent of familial risk, suggesting that genetic liability may not be a determinant of AAO. Previous studies have shown that precipitation of psychosis onset is highly probable due to the involvement of environmental risk factors, such as cannabis use and birth complications [74,75]. We found that positive FHP could not predict an earlier AAO, consistent with previous observations from family-based studies indicating that an earlier AAO in SZ cases may not be the result of an increased genetic liability within relatives, instead it may be determined by unique familial environmental influences for each individual and/or random developmental defects [43,45]. Other groups have reported an association between familial liability and an earlier AAO in FEP and SZ cases [36, 41,42], which could not be confirmed in our cohort possibly owing to the limited number of cases examined. We noted, however, that FEP cases had a later AAO compared to SZ cases which could be possibly attributed to the small number of females included in the FEP group that has been reported to develop psychosis at a later age than males [76,77]. Female cases were also more likely characterized by positive FHP, which is in line with previous studies [78,79].

More recently, analyses in a large UK clinical sample of individuals with psychotic disorders implicated poor social PA and younger AAO to treatment resistance [31]. We provide independent confirmation of the association between social PA aberrations in childhood and inadequate response to antipsychotic medication in FEP cases and treatment resistance in SZ cases. Together, the UK and our studies support the notion that social maladjustment in childhood may be seen as a risk marker for conversion to an early-onset psychosis with adverse clinical course (i.e., treatment resistance). Furthermore, in our substantially smaller sample of cases an earlier AAO was noticed among nonresponders to treatment, which is in accordance with the results of the UK study but requires further exploration since it falls short of statistical significance.

The current study postulates that the interplay of familial determinants, as well as socioeconomic environment could contribute to premorbid maladjustment among individuals who convert to psychosis. As transition to psychosis has been related to social PA dysfunction, the observed joint effect of familial risk and parental socioeconomic position on social PA could possibly imply the involvement of gene-by-environment interaction in SZ pathogenesis [66], even though it is stressed that familial liability do not only mirror increased genetic susceptibility and direct genetic implications could not be interrogated. The negative effect of social PA deficits on age at illness onset and treatment response may also be of important clinical relevance. Social PA impairment has been previously linked to early psychosis onset [31,32] and poor response to antipsychotic treatment [14,31,80] therefore early detection strategies targeting premorbid social adjustment in high-risk individuals could facilitate early intervention efforts aiming to delay illness onset and may also inform about potential resistance to treatment.

## Data Availability

The data that support the findings of this study are available from the corresponding author upon request. Restrictions apply to the availability of these data, which were used under license for this study. Data could be made available from the authors with the permission of Eginition Hospital Ethical Committee.

## Supplementary material

For supplementary material accompanying this paper visit http://dx.doi.org/10.1192/j.eurpsy.2020.41. click here to view supplementary material
