# Supplementary material for: Familial and socioeconomic contributions to premorbid functioning in psychosis: Impact on age at onset and treatment response
Source: Eur Psychiatry. 2020 Apr 29;63(1):e44. doi: 10.1192/j.eurpsy.2020.41 (PMC7355181; doi:10.1192/j.eurpsy.2020.41)
Supplement: Supplementary file 1 [file S0924933820000413sup001.docx]

**SUPPLEMENTARY TEXT**

**Determination of family history of psychosis**

Psychiatric diagnoses among biological relatives were recorded using the Family Interview for Genetic Studies (FIGS), which has been developed by the National Institute of Mental Health (NIMH) as a research oriented guide for systematically collecting information about relatives in family and genetic studies of psychiatric disorders. According to the FIGS, individuals are asked to provide lifetime diagnostic information about relatives in the pedigrees under investigation. Primarily, the interview focuses on first-degree relatives and offers the option to include information for affected second-degree relatives (extenders), provided that the proband or any available informant (i.e. parents, siblings, and spouse) could provide information for the presence of mental disorders in those relatives (i.e. grandparents, uncle or aunt, niece, nephew, half-sibs). The FIGS includes three parts: (a) the General Screening Questions which aim to gather general information about all known relatives in the pedigree, regardless of how distantly related (b) the Face Sheet, which is completed for each affected relative for whom the informant can provide information and (c) the Symptom Checklists, which are designed to obtain diagnostic details that help make possible a best estimate diagnosis. Symptom checklists for depression, psychosis, mania, alcohol or drug abuse and paranoid/schizoid/schizotypal personality are available. In the present study, the psychosis checklist was completed when there was evidence for a affective or non-affective psychotic disorder diagnosis among first or second degree relatives following the general screening questions, thus designating positive family history of psychosis (FHP). In cases where a relative had been diagnosed with a major psychiatric disorder, such as schizophrenia or bipolar disorder (type I), the completion of the depression, mania and psychosis symptom checklists were omitted (FIGS manual, 1992). The FIGS was administered by trained psychiatrists using information gathered from both the proband and his/her family members to ensure reliability.

**SUPPLEMENTARY TABLES**

| **Table S1:** Details on medication status at baseline for FEP and SZ cases | | |
| --- | --- | --- |
| **FEP patient group** | **Cases, n (%)** |  |
| drug-naïve | 60 (55.6) |  |
| treated for 1 day | 21 (19.4) |  |
| treated for 2-10 days | 27 (25.0) |  |
| treatment with 1 antipsychotic | 83 (76.9) |  |
| treatment with > 1 antipsychotics | 25 (23.1) |  |
| Non-remitters at follow-up | 36 (33.3) |  |
| **SZ patient group** | **Cases, n (%)** |  |
| treated for 1-2 years | 13 (12.5) |  |
| treated for 3-5 years | 11 (10.6) |  |
| treated for 6-10 years | 29 (27.9) |  |
| treated for > 10 years | 51 (49.0) |  |
| treatment with 1 antipsychotic | 67 (64.4) |  |
| treatment with > 1 antipsychotics | 37 (35.6) |  |
| Resistant cases (clozapine treated) | 34 (32.7) |  |
|  |  |  |

| **Table S2:** Association results between diagnostic status, gender and premorbid adjustment domains during childhood and early adolescence | | | | | | | | |
| --- | --- | --- | --- | --- | --- | --- | --- | --- |
|  | **FEP vs. SZ**  **(all cases, n=212)** | | | | **Females vs. Males**  **(all cases, n=212)** | | **Females vs. Males**  **(FEP cases, n=108)** | |
| **Childhood** | | **t-value** | **F (p-value)** | **t-value** | | **F (p-value)** | **t-value** | **F (p-value)** |
| Academic | | -1.26 | 1.59 (0.209) | -1.03 | | 1.05 (0.306) | -1.03 | 0.25 (0.617) |
| Social | | 1.11 | 1.22 (0.270) | 2.10 | | 4.41 (**0.037**) | 1.77 | 3.12 (0.081) |
| **Early Adolescense** | | **t-value** | **F (p-value)** | **t-value** | | **F (p-value)** | **t-value** | **F (p-value)** |
| Academic | | -1.70 | 2.89 (0.091) | -1.27 | | 1.62 (0.205) | -1.45 | 2.11 (0.150) |
| Social | | 0.40 | 0.16 (0.691) | 1.01 | | 1.01 (0.316) | 0.94 | 0.89 (0.348) |

**Table S3**: Results from linear regression analyses examining the main effects of family history of psychosis (FHP) and parental socioeconomic status (SES), as well as their interaction on PA domain scores.

|  | **FHP** | **SES** | **FHP x SES** |
| --- | --- | --- | --- |
| **Childhood PA** | **β (*p*-value)** | **β (*p*-value)** | **β (*p*-value*)** |
| Academic | 0.155 (**0.034**) | 0.131 (0.070) | 0.106 (0.218) |
| Social | 0.030 (0.683) | 0.039 (0.586) | 0.194 (**0.024**) |
| **Early adolescence PA** |  |  |  |
| Academic | 0.142 (0.062) | 0.104 (0.167) | 0.060 (0.508) |
| Social | 0.068 (0.363) | 0.027 (0.721) | 0.052 (0.570) |

*Results from multiple linear regression analyses, adjusting for gender, age, psychiatric site, diagnosis status (FEP, SZ) and the main effects of FHP, SES.

| **Table S4:** Association results between PA domain scores, age at onset and treatment response status in FEP and SZ patient groups | | | | | | | | | | | |  | |
| --- | --- | --- | --- | --- | --- | --- | --- | --- | --- | --- | --- | --- | --- |
|  | **FEP Cases** | | | **SZ Cases** | | **All Cases** | | | | **All Cases (adjusted*)** | | | |
|  | **OR**  **(95% CI)** | **p-value** | | **OR**  **(95% CI)** | **p-value** | | **OR**  **(95% CI)** | **p-value** | | | **OR**  **(95% CI)** | **p-value** | |
| **Childhood** |  | |  |  |  | |  |  | | |  | |  |
| **Academic** | 0.79  (0.48 - 1.30) | | 0.347 | 0.93  (0.59 - 1.45) | 0.740 | | 0.83  (0.61 - 1.14) | | 0.258 | | 0.84  (0.64 - 1.09) | | 0.270 |
| **Social** | 1.36  (0.84 - 2.19) | | 0.208 | 1.39  (0.85 - 2.27) | 0.188 | | 1.37  (0.99 - 1.89) | | 0.059 | | 1.37  (0.99 - 1.90) | | 0.057 |
| **Early adolescence** |  | |  |  |  | |  | |  | |  | |  |
| **Academic** | 1.09  (0.68-1.72) | | 0.729 | 0.87  (0.57-1.33) | 0.515 | | 0.97  (0.71-1.32) | | 0.821 | | 0.97  (0.71-1.32) | | 0.835 |
| **Social** | 1.25  (0.81-1.94) | | 0.322 | 1.12  (0.73-1.70) | 0.605 | | 1.18  (0.87-1.59) | | 0.286 | | 1.18  (0.87-1.60) | | 0.281 |
| **Age at onset (AAO)** | 0.50  (0.17 - 1.51) | | 0.221 | 0.43  (0.11 - 1.76) | 0.240 | | 0.47  (0.21 - 1.08) | | 0.075 | | 0.45  (0.19 - 1.08) | | 0.074 |
| * Adjusted for treatment status at baseline (drug naïve FEP, treated FEP for 1 day, treated FEP for 2-10 days, treated SZ for > 1 year) | | | | | | | | | | | |  | |
